# Computational evaluation of wire position using separate vertical wire technique and candy box technique for the fixation of inferior pole patellar fractures: a finite element analysis

**DOI:** 10.3389/fbioe.2024.1353901

**Published:** 2024-04-24

**Authors:** Wei Fan, Shunjie Dang, Xiaoqi Tan, Jinhui Liu, Yun-Kang Yang, Fei-Fan Xiang

**Affiliations:** ^1^ Department of Orthopedics, The Affiliated Hospital of Southwest Medical University, Luzhou, China; ^2^ Sichuan Provincial Laboratory of Orthopaedic Engineering, Luzhou, China; ^3^ Department of Dermatology, The Affiliated Hospital of Southwest Medical University, Luzhou, China; ^4^ State Key Laboratory of Quality Research in Chinese Medicine, Macau University of Science and Technology, Macau, China; ^5^ Department of Nuclear Medicine, Affiliated Hospital of Southwest Medical University, Luzhou, China

**Keywords:** separate vertical wire technique, candy box technique, finite element analysis, inferior pole patellar fractures, wire position

## Abstract

The separate vertical wire (SVW) technique and the improved candy box (CB) technique have been proposed for treating inferior pole patellar fractures. However, there is still a lack of clear explanation regarding the location of the wire passing through the patella. Five models of SVW techniques were established in different positions. Finite element analysis was then conducted to determine the optimal bone tunnel position for the SVW technique. Based on these findings, six groups of finite element models were created for CB techniques. The maximum displacement and stress on both the patella and steel wire were compared among these groups under 100-N, 200-N, 300-N, 400-N, and 500-N force loads. The results indicated that, in the SVW technique, the steel wire group near the fracture end of the longitudinal bone tunnel showed minimal displacement and stress on the patella when subjected to different forces. On the other hand, in the CB technique, both the patella and wire experienced minimal stress when a transverse bone tunnel wire was placed near the upper posterior aspect of patella. In conclusion, the SVW technique may require the bone tunnel wire to be positioned near the fractured end of the lower pole of the patella. On the other hand, in CB technique, the transverse bone tunnel wire passing through the patella may be close to its upper posterior aspect. However, further validation is necessary through comprehensive finite element analysis and additional biomechanical experiments.

## 1 Introduction

Inferior pole patellar fractures make up approximately 9.3%–22.4% of all patellar fractures (Zhu et al., 2020; Yan et al., 2023). These fractures are often comminuted and challenging to effectively reduce and fix due to the small size and concentrated stress in the fracture fragments (Oh et al., 2015). Among the internal fixation methods for patellar inferior pole fractures, the tension-band wiring combined with cerclage wiring (TBWC) technique is considered the gold standard for treating patellar fractures. However, when steel wires are inserted into the fractured comminuted area at the inferior pole, it can result in weak fixation and potential displacement of small bone fragments. This increases the risk of internal fixation failure and nonunion (Zhu et al., 2022; Fan et al., 2023).

The SVW technique, proposed by Yang et al., in 2003, is a specialized internal fixation technique used for treating inferior pole patellar fractures. Biomechanical experiments and clinical studies have been conducted on this technique, revealing that out of 29 patients, two experienced complications such as wire loosening (Yang and Byun, 2003). Since then, there have been continuous explorations using the SVW technique and its modified versions for treating inferior pole patellar fractures. In 2014, Song et al. combined the SVW technique with circumferential wiring (SVWC) to treat these fractures in osteoporotic patients. They conducted biomechanical studies on cadaver specimens using both the original and improved techniques, test results showed that after improvement, the average failure load increased from 216N to 324N. However, in clinical studies involving four patients (Song et al., 2014), complications such as wire fracture were still observed. In 2018, He et al. further modified SVW (MSVW) by replacing encircling steel wire to bone-penetrating tunnel steel wire. Clinical research results indicated that this new technique addressed most previous issues but still had occurrences of complications like wire loosening (He et al., 2018).

Our team initially introduced the CB technique as an improvement to the SVWC technique. In this new approach, we replace the circumferential wire of the SVWC technique with two transverse bone tunnel wires positioned at the upper/middle 1/3 of the patella. We then conducted finite element analysis and biomechanical testing, which confirmed that the CB technique performs exceptionally well under different loads (Fan et al., 2023). However, there is currently no definitive explanation for the optimal positions of vertical bone tunnel steel wires in SVW technology and horizontal bone tunnel steel wires in CB technology.

Considering the treatment effects of previous and improved versions of SVW techniques, as well as unresolved complications like wire loosening, We hypothesize that the varying positions of the patellar bone tunnel wire may impact the biomechanical environment, potentially leading to different clinical outcomes. To investigate this further, we established finite element models for 5 groups of SVW techniques with different positions. Through finite element analysis, we identified the best position for the vertical bone tunnel wire in SVW technique. Using this optimal position as a reference, we developed models for 6 groups of CB techniques and used finite element analysis to calculate potential optimal positions for transverse bone tunnel wires passing through the patella. This research may offer practical guidance for surgical operations.

## 2 Materials and methods

### 2.1 Development of candy box technology

**FIGURE 1:**
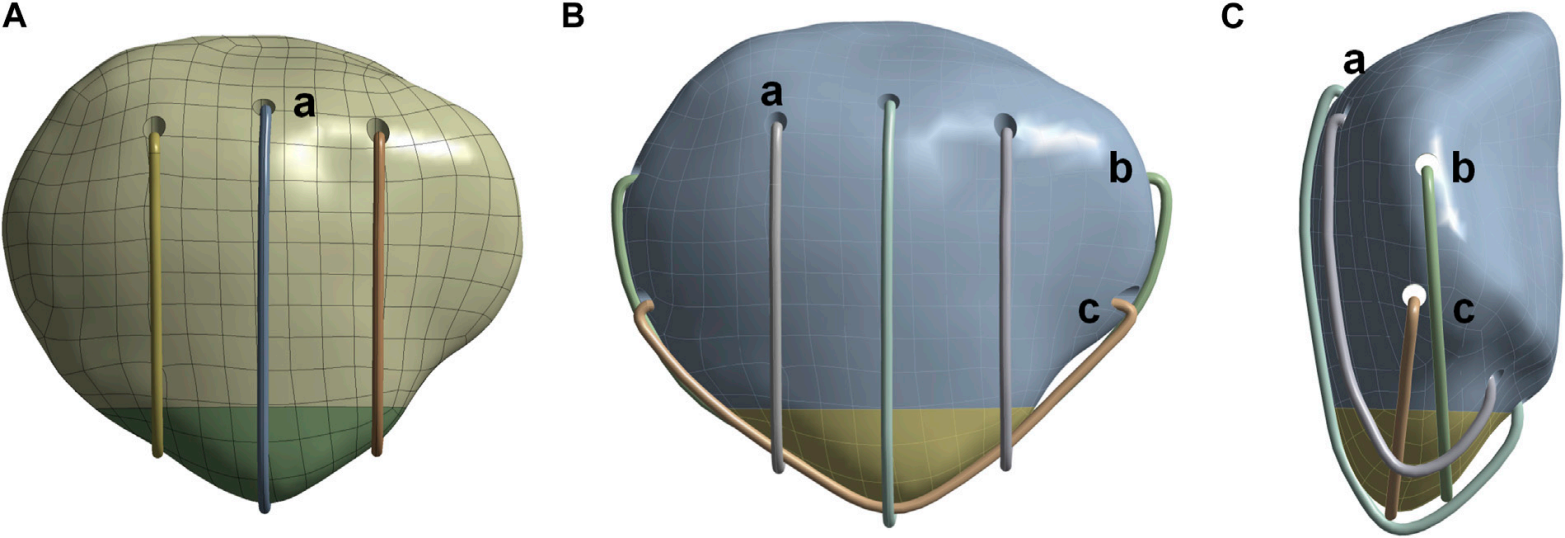
Schematic diagram of separate vertical wire and candy box technology. **(A)** Front view of separate vertical wire technology. **(B)** Front view of candy box technology. **(C)** Side view of candy box technology. **(a)** Three separate vertical steel wires. **(b)** Upper 1/3 steel wires of the patellar tunnel. **(c)** Middle 1/3 steel wires of the patellar tunnel.

In previous studies, we have enhanced the MSVW technique by combining separate vertical wire fixation with proximal patellar wire double ligation. The distribution of the improved steel wire is similar to that of a candy box, Therefore, it is called Candy Box (CB) technology. The key improvements include fixing the fractured end with three separate vertical steel wires and creating bone tunnels in the upper and middle one-third of the patella. Then, the fixed wire around the periphery of the patella was replaced with two wires on the side of the patella through the bone tunnel (Figure 1). Our team has previously confirmed both biomechanical advantages and potential clinical benefits associated with CB technology (Fan et al., 2023).

### 2.2 Initial design of vertical bone tunnel steel wire positions in SVW technique

**FIGURE 2:**
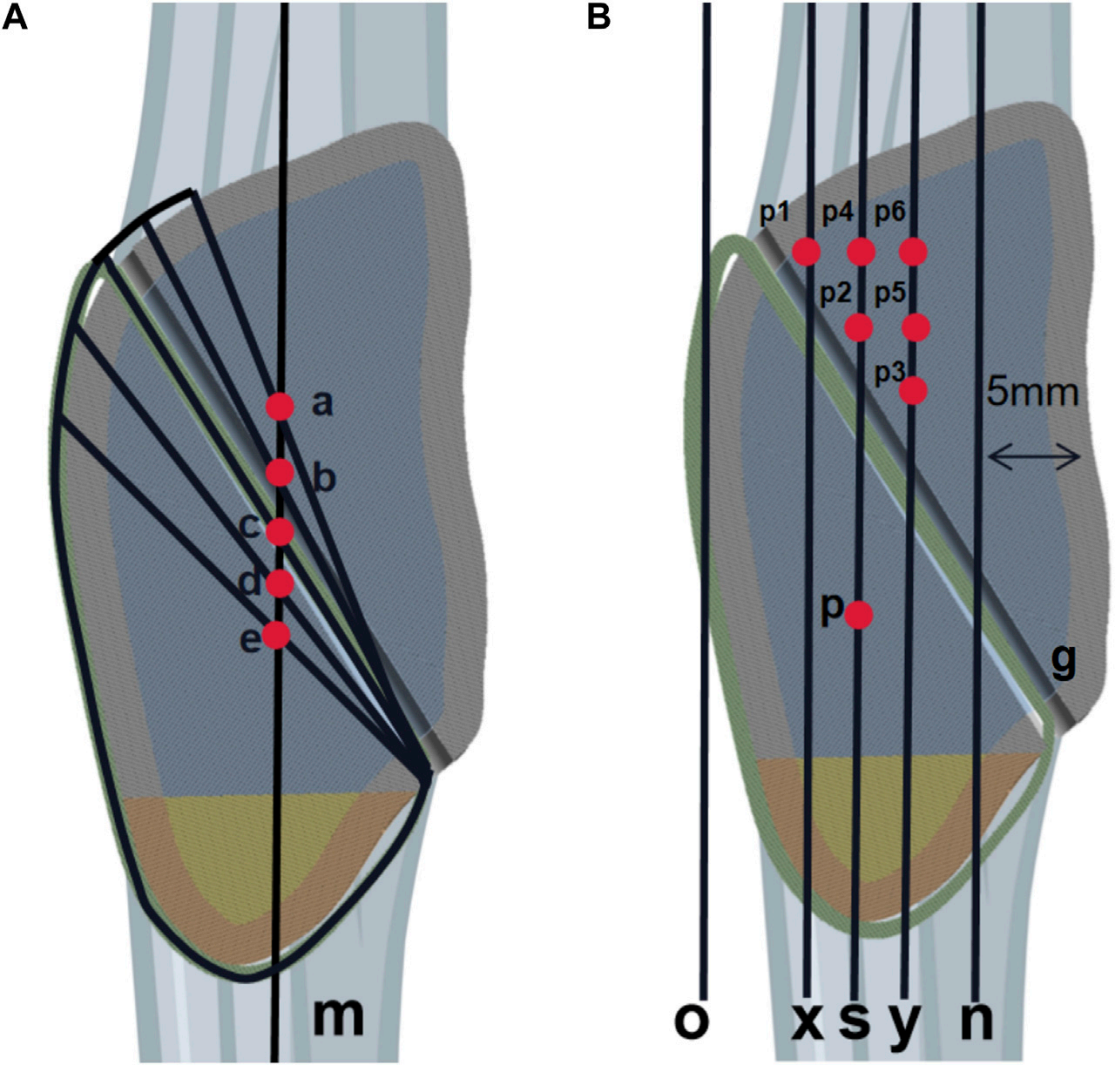
Schematic diagram of the research plan. **(A)** The first part of the diagram shows five vertical steel wires positioned at different locations. Line m is the midline of the patella. The intersection point between the starting point behind the fracture end and the stop point of the quadriceps tendon with line m is referred to as group c. Group b and group d intersect with line m at positions 2 mm above and below, respectively, under the same starting point condition. Similarly, group a and group e intersect with line m at positions 4 mm above and below, respectively. In total, there are five groups in the first part. **(B)** In Part 2, the diagram illustrates the positions of two horizontal steel wires. Line n represents a vertical line located 5 mm behind the articular surface of the patella, Line o represents a vertical line directly in front of the patella, Line s represents the midpoint line between lines o and n, Lines x and y represent vertical lines positioned 2 mm in front and behind line s, respectively. Steel wire g is considered to be in an optimal position based on Part 1 of the study. Point p is situated below line s, with a distance of 4 mm from steel wire g. Points p1, p2, and p3 are taken at positions that are 2 mm away from steel wire g along lines x, s, and y respectively, Points p4 and p5 are located above points p2 and p3 with an interval of 2 mm, Point p6 is positioned above point p5 with a distance of 2 mm. Overall, there are six groups included in Part 2.

To construct the model of SVW technique for different positions of bone tunnels, we begin by drawing a midline “m” on the lateral view section of the patella. To ensure proper fixation of the entire distal fracture fragment with SVW technique, we start from the posterior aspect of the fracture end and draw a line up to the anterior attachment point of quadriceps tendon on the patella. The intersection point between this line and “m” is labeled as group “c.” Using this same starting point, groups “b” and “d” are obtained by intersecting points 2 mm above and below line “m,” respectively. Similarly, groups “a” and “e” are obtained by intersecting points 4 mm above and below line “m,” respectively (Figure 2A). In total, there are five groups (a, b, c, d, e) for internal fixation in this part.

### 2.3 Initial design of transverse bone tunnel steel wire positions in CB technique

The research on the transverse bone tunnel wire position for CB technology was conducted after determining the optimal position for SVW technology. The optimal vertical position is represented by wire g. Line n is a vertical line located 5 mm front the posterior articular surface of the patella, while line o is a vertical line directly in front of the patella. The midline between lines o and n is represented by line s. Lines x and y are positioned 2 mm in front and behind line s, respectively. Point p is located on line s with a 4 mm gap from wire g. Points p1, p2, and p3 are taken at positions 2 mm away from wire g along lines x, s, and y respectively. Points p4 and p5 are positioned above points p2 and p3 with a 2 mm gap between them. Point p6 is placed above point p5 with another 2 mm gap. This section includes six sets of internal fixation: p1, p2, p3, p4, p5, and p6 (Figure 2B).

### 2.4 Establishment of initial 3D model

A healthy adult volunteer underwent a scan of the entire knee joint using a 128-layer spiral computed tomography scanner (SIMENS SENSATION 128, resolution: 512*512 pixels). The resulting image data was saved in DICOM format and imported into Mimics 21 modeling software. From there, the patellar region was chosen and the patellar polyline was extracted to generate a three-dimensional model of both the patella and inferior pole patellar fracture. This model was then processed for denoising, encapsulation, and smoothing using Geomagic Studio 2021 software.

### 2.5 Establishment and evaluation of internal fixation models

The established three-dimensional fracture model was inputted into the ANSYS WORKBENCH2021 finite element analysis software. The elastic modulus and Poisson’s ratio of bones and steel wires in the model were defined based on previous research (as shown in Table 1) (Du et al., 2022). Frictional contact was set between the periphery of the patella and bone tunnel wire, as well as between the patella and vertical wire, with a friction coefficient of 0.2 (Zhu et al., 2022). The friction coefficient of the fractured end of the inferior pole of the patella was set to 0.45 (Chang et al., 2018; Demirtaş and Kati, 2023).

**TABLE 1:** Model material parameters.

Name of the material	Elastic modulus (MPa)	Poisson ratio
Cortical bone	10,000	0.30
Cancellous bone	840	0.29
Steel wire	100,000	0.29
Kirschner wire	200,000	0.30

**FIGURE 3:**
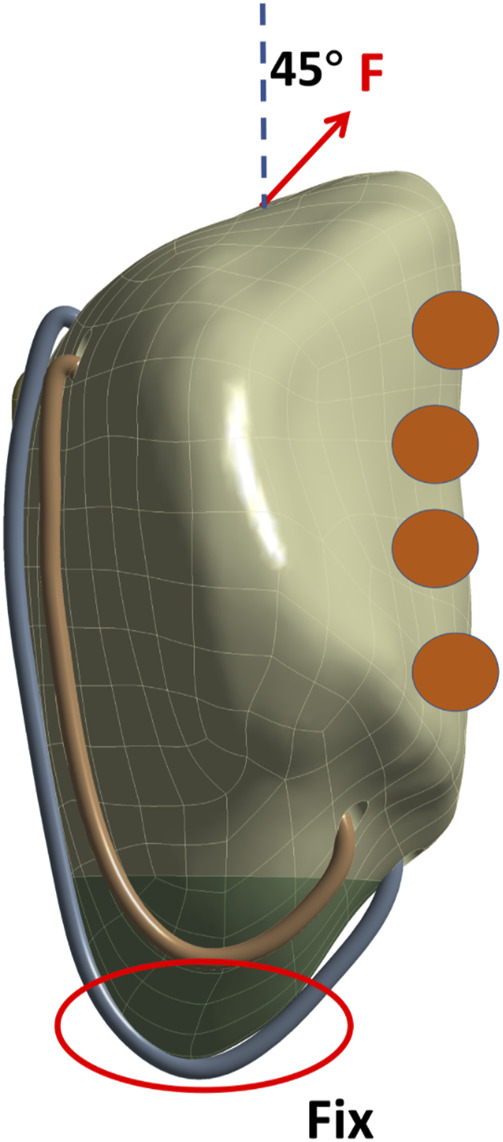
Schematic diagram of patella loading. The inferior pole of the patella is bound and constrained, the upper pole is loaded, and the arrow indicates the stretching direction. The orange circles represent the support of the medial and lateral condyles of the femur to the patella.

Models for five SVW techniques and six CB techniques were established according to the research plan and initial design. We applied load on the upper end of the patella while constraining its lower end. Compression Only Support software was used behind the patella to simulate internal and external condyles of femur, with an angle of 45° relative to the long axis of patella to simulate forces on knee joint (Chen et al., 2019) (Figure 3).

### 2.6 Evaluation indices

We analyzed the stress and displacement distribution of the patella and internal fixation using the solution module of ANSYS WORKBENCH 2021 software. The results were represented in Nephogram format. Additionally, we calculated the maximum stress and displacement values for both the patella and internal fixation, presenting them in Histogram form. This analysis is conducted under loads of 100-N, 200-N, 300-N, 400-N, and 500-N for five models of SVW technology and six models of CB technology.

## 3 Results

### 3.1 Evaluation of the ideal placement of vertical steel wires through bone tunnels

**FIGURE 4:**
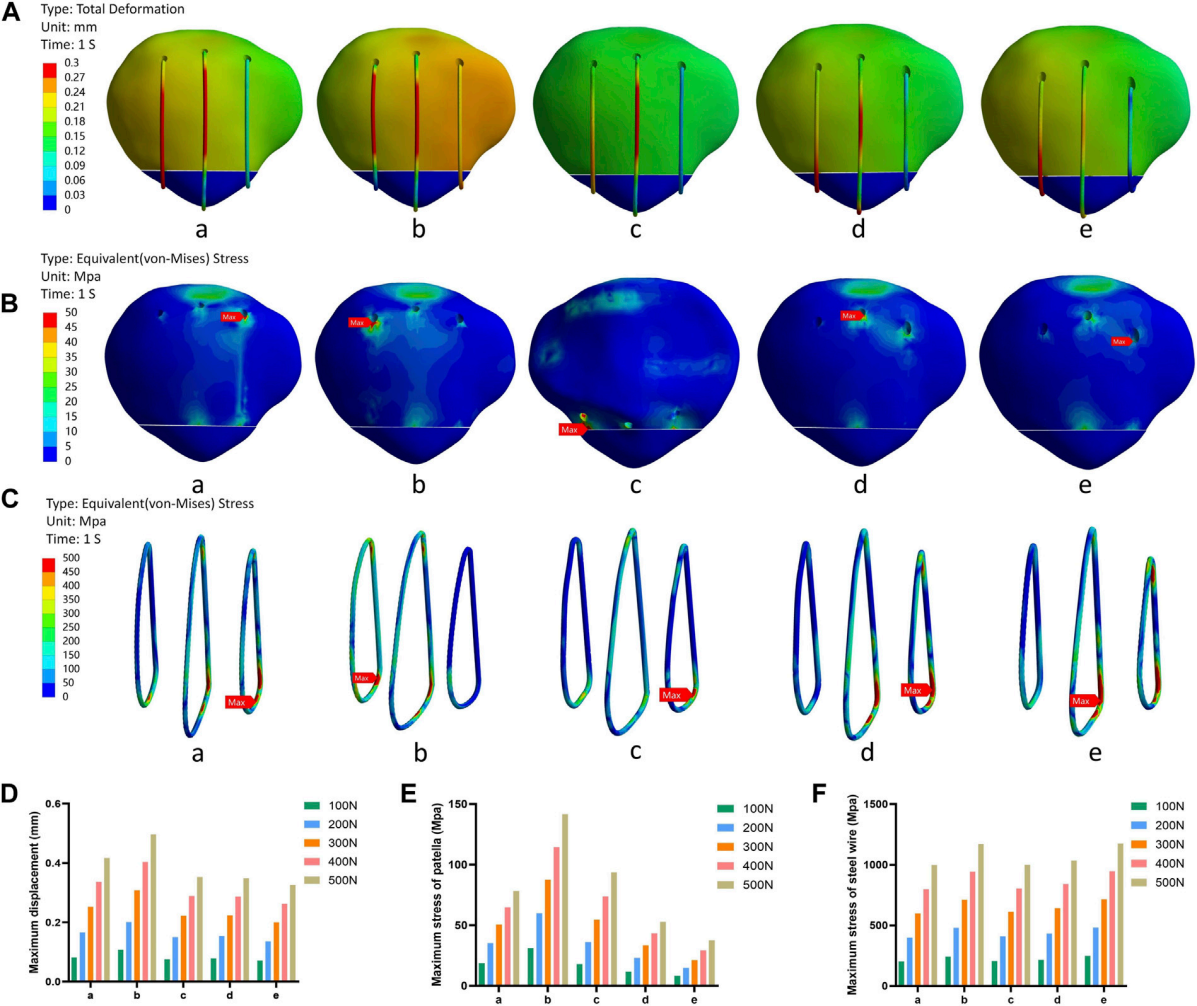
The schematic diagram of the research results in the first part. **(A)** Displacement nephogram under 500N force. **(B)** Stress nephogram of patella under 500N force. **(C)** Stress nephogram of steel wire under 500N force. **(D)** Histogram of the maximum displacement of different groups under different forces. **(E)** Histogram of the maximum stress of patella under different forces. **(F)** Histogram of the maximum stress of steel wire under different forces. Note: In this context, “a,b,c,d,e” represent different positions along the vertical wire.

The results of the finite element analysis indicated that group e had the lowest maximum displacement and patellar stress values under various forces. However, it also exhibited higher wire stress values (Figure 4).

### 3.2 Evaluation of the ideal placement for the transverse patellar tunnels

**FIGURE 5:**
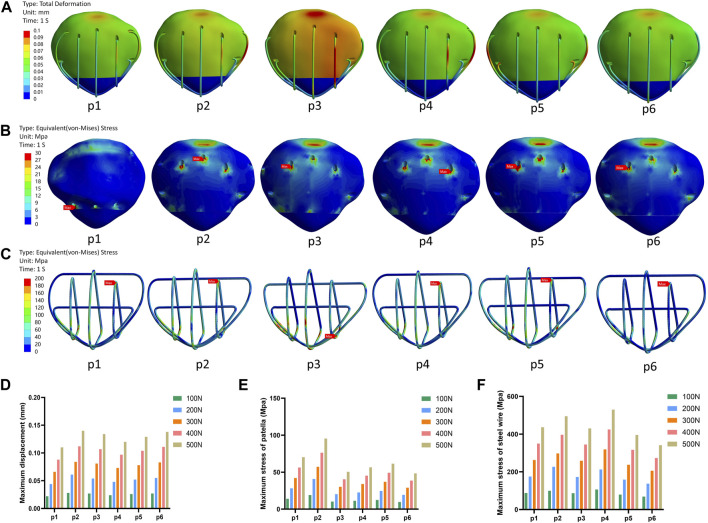
The schematic diagram of the research results in the second part. **(A)** Displacement nephogram under 500N force. **(B)** Stress nephogram of patella under 500N force. **(C)** Stress nephogram of steel wire under 500N force. **(D)** Histogram of the maximum displacement of different groups under different forces. **(E)** Histogram of the maximum stress of patella under different forces. **(F)** Histogram of the maximum stress of steel wire under different forces. Note: In this context, p1, p2, p3, p4, p5, and p6 respectively represent different positions of the upper 1/3 of the wire.

The finite element analysis results indicated that the p6 group exhibited smaller values of wire stress and patellar stress under different forces, while the p1 group exhibited smaller maximum displacement values (Figure 5).

## 4 Discussion

This study evaluated the position of steel wires in two techniques, SVW and CB, for treating fractures in the inferior pole of the patella. The findings suggest that in SVW technology, placing the steel wire group near the fracture end of the longitudinal bone tunnel results in minimal displacement and stress on the patella under various forces. Additionally, in CB technology, positioning the transverse bone tunnel line near the posterior upper part of the patella leads to reduced stress on both the patella and metal wire.

Fractures in the inferior pole of the patella can cause damage to the extensor mechanism and disrupt normal anatomical and biomechanical relationships in the knee joint (O'Donnell et al., 2021). Therefore, it is essential to treat these fractures correctly in order to restore stability, integrity, and function (Yang et al., 2017; Huang et al., 2021). The SVW technique, along with its modified techniques, has become an important method for treating such fractures (Fan et al., 2023; Yan et al., 2023). However, the placement of the steel wire in the patellar bone tunnel has always been a significant challenge for surgeons during actual operations. After reviewing the literature, we discovered that Yang and Song’s SVW technique places the vertical steel wire puncture point near the quadriceps tendon, similar to group a or b in this study (Yang and Byun, 2003; Song et al., 2014). On the other hand, He’s MSVW technique in 2018 positions the longitudinal steel wire closer to the fractured end of patella, resembling group d or e mentioned previously(He et al., 2018). These clinical studies have reported various complications such as different degrees of wire loosening. Consequently, we hypothesize that different positions of patellar bone tunnel steel wires may result in distinct biomechanical environment and potentially lead to diverse clinical outcomes.

In the first part of our research, we observed that Group e had the smallest maximum displacement and minimum patellar stress values under different forces. However, it also had higher wire stress values. This could be attributed to a phenomenon called “stress shielding,” where components with higher elastic modulus bear more load when connected in parallel (Amirouche et al., 2017). As a result, they provide a stress and strain shielding effect on components with lower elastic modulus. Consequently, this leads to significantly higher wire stress compared to patellar stress at the same location (Pettersen et al., 2009; Garabano et al., 2022). Additionally, the smaller fixation range of the wires near the fracture end indirectly causes a more concentrated stress on them. However, in cases of inferior pole patellar fractures, a smaller fixation range can shift the center of gravity towards “inferior central fixation,” which may explain why Group e shows better biomechanical characteristics. Therefore, based on this part of the study, we tentatively consider that for SVW technique, positioning vertical bone tunnels near the fracture end may be safer.

In a previous biomechanical study, it was found that damaging the anterior patellar cortex during media patellofemoral ligament (MPFL) reconstruction surgery can increase the risk of patellar fracture (Bonazza et al., 2018). As a result, when deciding on the placement of the middle 1/3 steel wire passing through the transverse patellar tunnel in CB technology, we opted for a p-point that is located far from the anterior cortex. Additionally, during the finite element modeling process, we discovered a potential risk of the wire passing directly through the joint cavity when it goes through the transverse bone tunnel within 5 mm behind the patella. To address this issue, we decided to reserve a range of 5 mm behind the patella (line n). Additionally, if two bone tunnel wires are too close to each other, it may lead to convergence and surgical failure during actual operation. Furthermore, the first part of this study revealed that steel wires positioned closer to the fracture end may result in better internal fixation. As a consequence, there are fewer available bone tunnels for insertion in the middle 1/3 of the patella. Therefore, we only select the p-point located far from the anterior cortex of the patella and 4 mm away from the vertical steel wire as an insertion point for the middle 1/3 of the steel wire. Conversely, there is more space remaining in the upper 1/3 of the patella. Consequently, our study focused solely on studying CB technology’s transverse bone tunnel on this specific area.

The results of the second part of this study indicate that the steel wire experienced minimal stress in both the steel wire and patellar when passing through position p6, which aligns with previous biomechanical research (Wierer et al., 2022). Additionally, it was observed that p1 had the smallest maximum displacement value under different forces. However, all displacement values recorded in this study were well below the failure criterion (3 mm) for transverse bone tunnels in the patella (Patel et al., 2000). Based on these findings, we tentatively suggest placing the tunnel above and behind the patella, as far away from its anterior cortex as possible. This approach may create a more favorable biomechanical environment for the patella and potentially enhance clinical outcomes.

This study has several limitations. Firstly, our model does not accurately simulate the motion of the knee joint. Secondly, we only evaluated forces at a 45-degree angle of the patella and assessed force processes for transverse fractures at the lower pole of the patella. Additionally, we did not conduct further biomechanical experiments or clinical studies. Therefore, future research should focus on establishing comprehensive finite element models for different types of fractures from various force angles. Finally, long-term clinical studies are necessary to validate our findings.

## 5 Conclusion

The SVW technique may require the bone tunnel wire to be near the fracture end close to the lower pole of the patella. On the other hand, in CB technique, the transverse bone tunnel wire passing through the patella may be close to its upper posterior aspect. These positioning differences could potentially improve biomechanical environment and lead to better clinical outcomes. However, further validation is required through comprehensive finite element analysis and additional biomechanical experiments.

## Data Availability

The original contributions presented in the study are included in the article/Supplementary material, further inquiries can be directed to the corresponding authors.

## References

[B1] Amirouche F. Solitro G. F. Walia A. Gonzalez M. Bobko A. (2017). Segmental acetabular rim defects, bone loss, oversizing, and press fit cup in total hip arthroplasty evaluated with a probabilistic finite element analysis. Int. Orthop. 41 (8), 1527–1533. 10.1007/s00264-016-3369-y 28012048

[B2] Bonazza N. A. Lewis G. S. Lukosius E. Z. Roush E. P. Black K. P. Dhawan A. (2018). Effect of transosseous tunnels on patella fracture risk after medial patellofemoral ligament reconstruction: a cadaveric study. Arthroscopy 34 (2), 513–518. 10.1016/j.arthro.2017.08.267 29100765

[B3] Chang C. W. Chen Y. N. Li C. T. Chung Y. H. Chang C. H. Peng Y. T. (2018). Role of screw proximity in the fixation of transverse patellar fractures with screws and a wire. J. Orthop. Surg. (Hong Kong) 26 (3), 230949901878970. 10.1177/2309499018789705 30037293

[B4] Chen C. H. Chen Y. N. Li C. T. Chang C. W. Chang C. H. Peng Y. T. (2019). Roles of the screw types, proximity and anterior band wiring in the surgical fixation of transverse patellar fractures: a finite element investigation. BMC Musculoskelet. Disord. 20 (1), 99. 10.1186/s12891-019-2474-7 30832645 PMC6399979

[B5] Demirtaş Y. Katı Y. A. (2023). A novel patella fracture fixation technique: finite element analysis. Archives Orthop. trauma Surg. 143 (8), 5105–5115. 10.1007/s00402-023-04910-1 37233796

[B6] Du B. Ma T. Bai H. Lu Y. Xu Y. Yang Y. (2022). Efficacy comparison of Kirschner-wire tension band combined with patellar cerclage and anchor-loop plate in treatment of inferior patellar pole fracture. Front. Bioeng. Biotechnol. 10, 1010508. 10.3389/fbioe.2022.1010508 36324895 PMC9618880

[B7] Fan W. Liu J. Tan X. Wei D. Yang Y. Xiang F. (2023). Candy box technique for the fixation of inferior pole patellar fractures: finite element analysis and biomechanical experiments. BMC Musculoskelet. Disord. 24 (1), 835. 10.1186/s12891-023-06946-1 37872511 PMC10594795

[B8] Fan W. Xiao Y. Xiang F. Yang Y. (2023). Research progress of inferior-pole patellar fracture: a systematic review. Asian J. Surg. 46 (6), 2450–2451. 10.1016/j.asjsur.2022.12.057 36577583

[B9] Garabano G. Rodriguez J. Perez Alamino L. Pesciallo C. A. Del Sel H. Lopreite F. (2022). Stress shielding in total knee replacements: comparative analysis between titanium and all-polyethylene bases at 10 years follow-up. J. Orthop. 34, 276–281. 10.1016/j.jor.2022.09.007 36158038 PMC9493296

[B10] He S. Huang X. Yan B. Zhu J. Bao N. Zhao J. (2018). Modified technique of separate vertical wiring for the fixation of patellar inferior Pole fracture. J. Orthop. trauma 32 (4), e145–e150. 10.1097/BOT.0000000000001080 29557940

[B11] Huang W. Wu T. Wei Q. Peng L. Cheng X. Gao G. (2021). Suture repair of patellar inferior pole fracture: transosseous tunnel suture compared with anchor suture. Exp. Ther. Med. 22 (3), 998. 10.3892/etm.2021.10430 34345280 PMC8311267

[B12] O'Donnell R. Lemme N. J. Marcaccio S. Walsh D. F. Shah K. N. Owens B. D. (2021). Suture anchor versus transosseous tunnel repair for inferior Pole patellar fractures treated with partial patellectomy and tendon advancement: a biomechanical study. Orthop. J. sports Med. 9 (8), 23259671211022245. 10.1177/23259671211022245 34423057 PMC8371734

[B13] Oh H. K. Choo S. K. Kim J. W. Lee M. (2015). Internal fixation of displaced inferior pole of the patella fractures using vertical wiring augmented with Krachow suturing. Injury 46 (12), 2512–2515. 10.1016/j.injury.2015.09.026 26482481

[B14] Patel V. R. Parks B. G. Wang Y. Ebert F. R. Jinnah R. H. (2000). Fixation of patella fractures with braided polyester suture: a biomechanical study. Injury 31 (1), 1–6. 10.1016/s0020-1383(99)00190-4 10716043

[B15] Pettersen S. H. Wik T. S. Skallerud B. (2009). Subject specific finite element analysis of stress shielding around a cementless femoral stem. Clin. Biomech. (Bristol, Avon) 24 (2), 196–202. 10.1016/j.clinbiomech.2008.11.003 19103468

[B16] Song H. K. Yoo J. H. Byun Y. S. Yang K. H. (2014). Separate vertical wiring for the fixation of comminuted fractures of the inferior pole of the patella. Yonsei Med. J. 55 (3), 785–791. 10.3349/ymj.2014.55.3.785 24719149 PMC3990064

[B17] Wierer G. Winkler P. W. Pomwenger W. Plachel F. Moroder P. Seitlinger G. (2022). Transpatellar bone tunnels perforating the lateral or anterior cortex increase the risk of patellar fracture in MPFL reconstruction: a finite element analysis and survey of the International Patellofemoral Study Group. Knee Surg. sports Traumatol. Arthrosc. 30 (5), 1620–1628. 10.1007/s00167-021-06682-w 34333671

[B18] Yan S. G. Li D. Cui Y. Hua X. Hemmann P. Schmidutz F. (2023). Management of comminuted inferior patellar pole fractures with cerclage-wire-augmented separate vertical wiring: a retrospective clinical study. Archives Orthop. trauma Surg. 143 (1), 247–254. 10.1007/s00402-021-04034-4 34232348

[B19] Yang K. H. Byun Y. S. (2003). Separate vertical wiring for the fixation of comminuted fractures of the inferior pole of the patella. J. bone Jt. Surg. Br. volume 85 (8), 1155–1160. 10.1302/0301-620x.85b8.14080 14653599

[B20] Yang X. Wu Q. Lai C. H. Wang X. (2017). Management of displaced inferior patellar pole fractures with modified tension band technique combined with cable cerclage using Cable Grip System. Injury 48 (10), 2348–2353. 10.1016/j.injury.2017.07.013 28733044

[B21] Zhu W. Xie K. Li X. Li L. Yang J. Xu L. (2020). Combination of a miniplate with tension band wiring for inferior patellar pole avulsion fractures. Injury 51 (3), 764–768. 10.1016/j.injury.2020.01.028 32005322

[B22] Zhu W. Xu L. Xie K. Li X. Zhang X. Fang S. (2022). Design and validation of a smile-necklace plate for treating inferior patellar Pole avulsion fractures: a review and hypothesis. Orthop. Surg. 14 (11), 2799–2808. 10.1111/os.13490 36125193 PMC9627049

